# Infodemiological data of West-Nile virus disease in Italy in the study period 2004–2015

**DOI:** 10.1016/j.dib.2016.10.022

**Published:** 2016-11-02

**Authors:** Nicola Luigi Bragazzi, Susanna Bacigaluppi, Chiara Robba, Anna Siri, Giovanna Canepa, Francesco Brigo

**Affiliations:** a School of Public Health, Department of Health Sciences (DISSAL), University of Genoa, Genoa, Italy; b UNESCO CHAIR “Anthropology of Health – Biosphere and Healing System”, University of Genoa, Genoa, Italy; c Department of Mathematics (DIMA), University of Genoa, Genoa, Italy; d Department of Neuroscience, Rehabilitation, Ophthalmology, Genetics, Maternal and Child Health, Section of Psychiatry, University of Genoa, Genoa, Italy; e Galliera Hospital, Department of Neurosurgery, Genoa, Italy; f Neurosciences Critical Care Unit, Addenbrooke׳s Hospital, Cambridge University, Cambridge University Hospitals Trust, Cambridge, United Kingdom; g Department of Neurosciences, Biomedical, and Movement Sciences, University of Verona, Italy; h Department of Neurology, Franz Tappeiner Hospital, Merano, Italy

**Keywords:** Google Trends, Infodemiology and infoveillance, West-Nile virus disease

## Abstract

Google Trends (GT) was mined from 2004 to 2015, searching for West-Nile virus disease (WNVD) in Italy. GT-generated data were modeled as a time series and were analyzed using classical time series analyses. In particular, correlation between GT-based Relative Search Volumes (RSVs) related to WNVD and “real-world” epidemiological cases in the same study period resulted *r*=0.76 (*p*<0.0001) on a monthly basis and *r*=0.80 (*p*<0.0001) on a yearly basis. The partial autocorrelation analysis and the spectral analysis confirmed that a 1-year regular pattern could be detected. Correlation between GT-based RSVs related to WNVD yielded a *r*=0.54 (*p*<0.05) on a regional basis. Summarizing, GT-generated data concerning WNVD well correlated with epidemiology and could be exploited for complementing traditional surveillance.

**Specifications Table**

**Table:** 

Subject area	*Epidemiology*
More specific subject area	*Digital epidemiology*
Type of data	*Table and graphs*
How data were acquired	*Outsourcing of Google Trends website and of the Italian National Health Institute (ISS) site concerning West-Nile virus disease*
Data format	*Raw, analyzed*
Experimental factors	*Google Trends search volumes were obtained through heat-maps*
Experimental features	*Validation of Google Trends-based data with “real-world” data taken from the Italian National Health Institute (ISS) was performed by means of correlational analysis. Further, autocorrelation and partial autocorrelation analyses and regressions were carried out.*
Data source location	*Italy*
Data accessibility	*Data are within this article*

**Value of the data**

• To the best of our knowledge, this is the first thorough quantitative analysis of West-Nile virus disease related web activities.
• The analyses presented in this data article show that Google Trends-generated data concerning the West-Nile virus disease well correlated with epidemiology in Italy.
• This analysis could be extended in other countries, in order to replicate the current findings in other settings and contexts.
• These data could be further mathematically and statistically refined for designing an approach for complementing traditional surveillance of the West-Nile virus disease.

## 1 Data

**Fig. 1:**
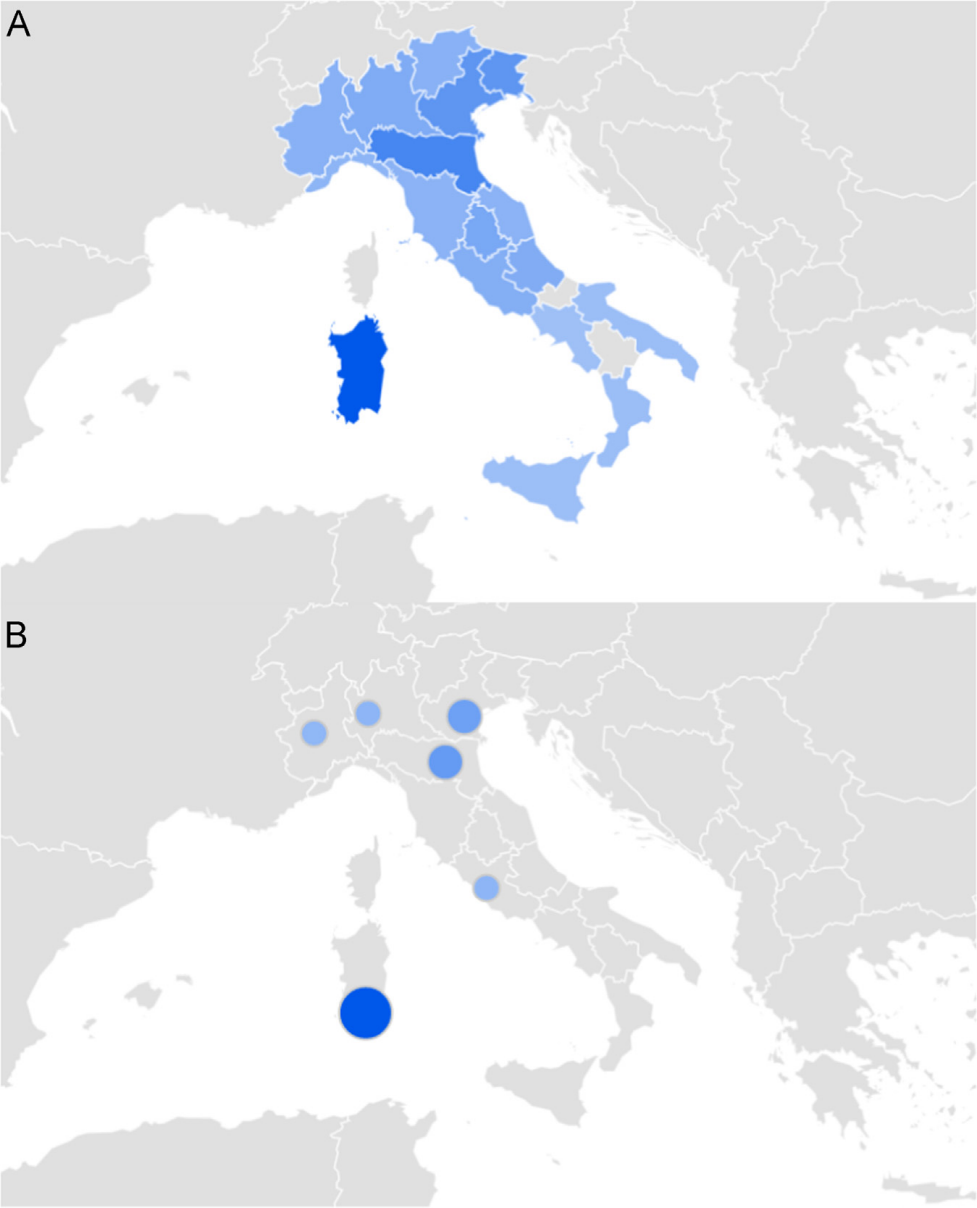
Digital interest for West-Nile virus disease in Italy at regional (A) and town (B) level, as captured by Google Trends.

**Fig. 2:**
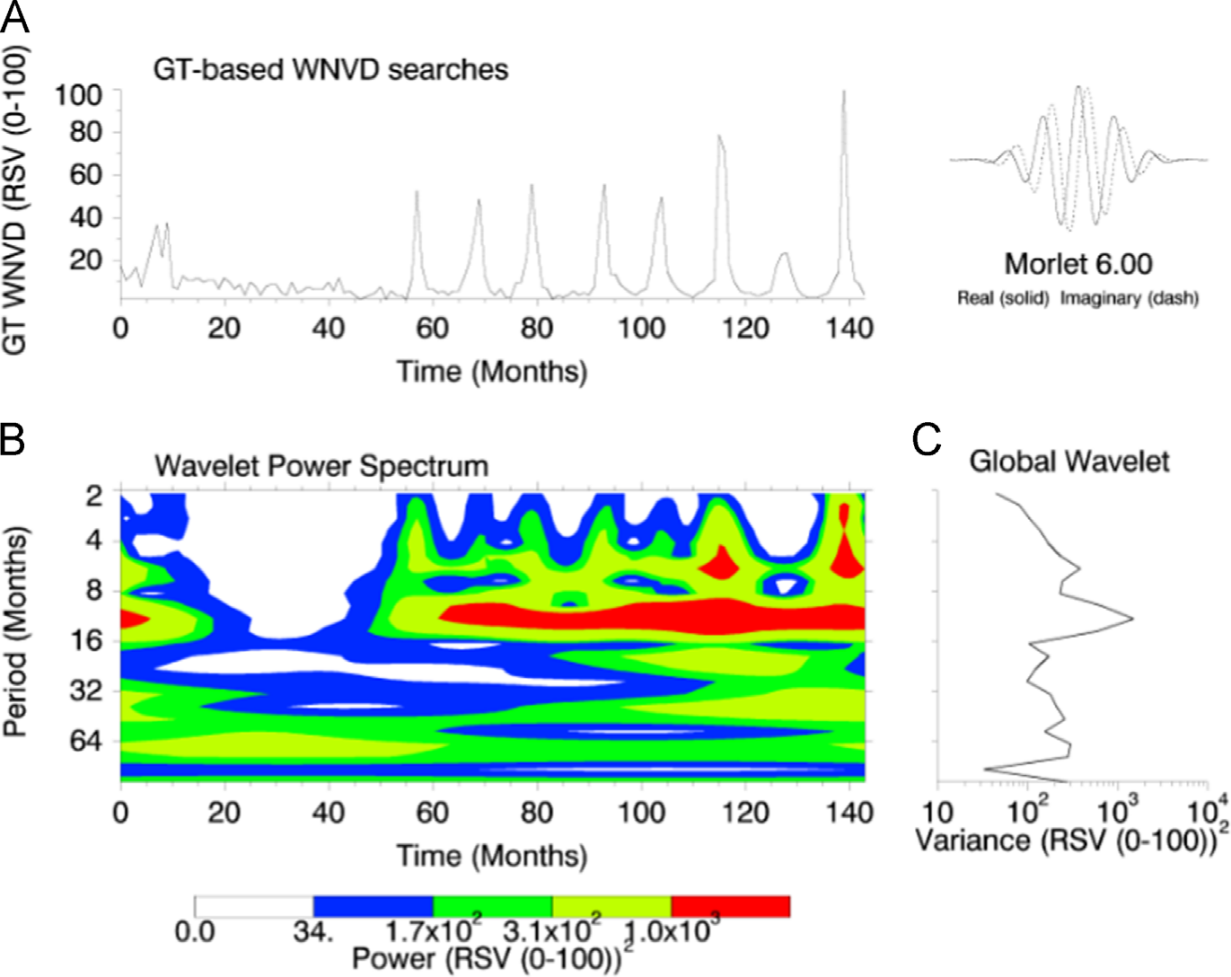
(a) GT-based West-Nile virus disease related web-searches. (b) The wavelet power spectrum. The contour levels are chosen so that 75%, 50%, 25%, and 5% of the wavelet power is above each level, respectively. A statistically significant regular 1-year pattern can be detected. (c) The global wavelet power spectrum.

**Fig. 3:**
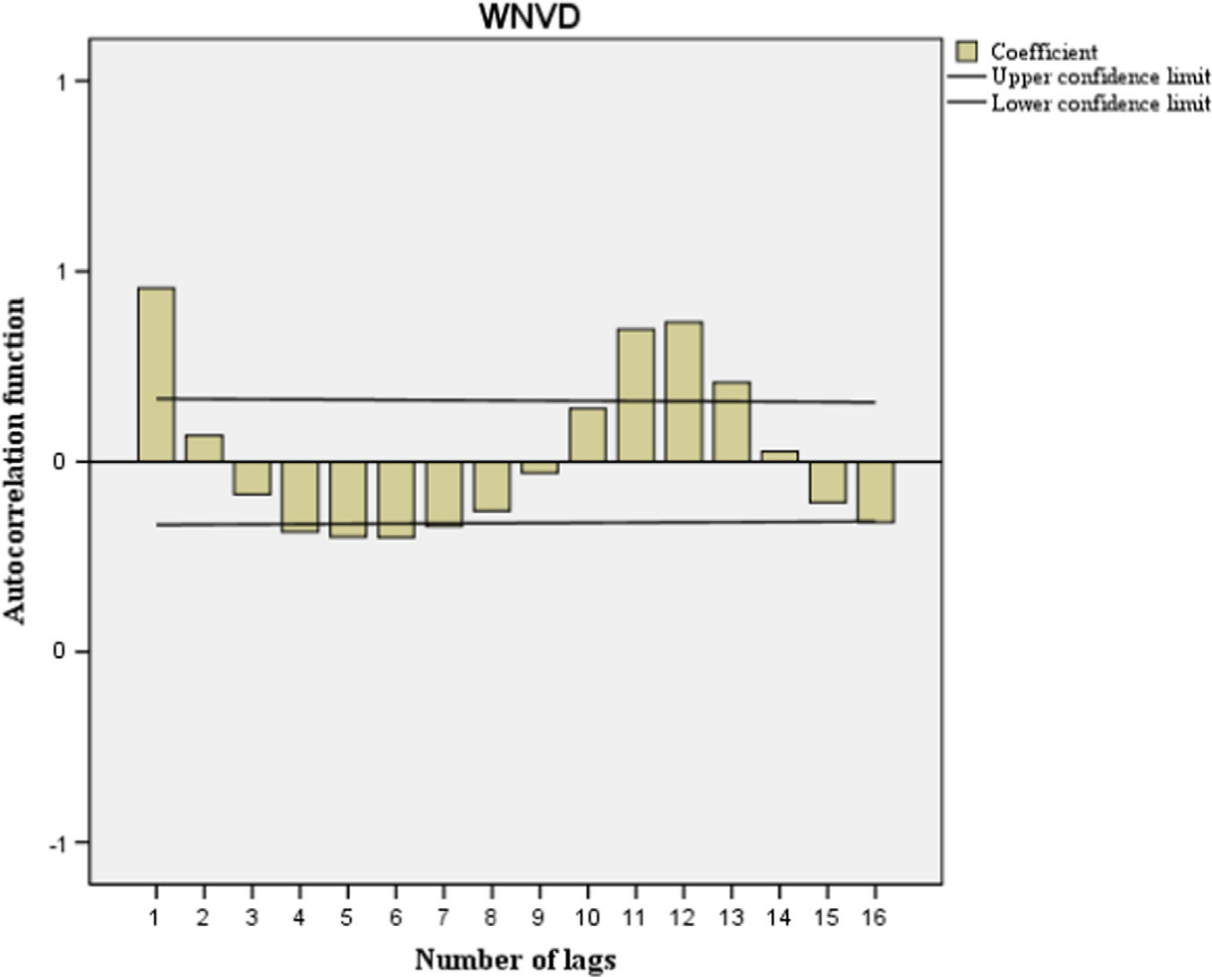
Google Trends-generated data concerning the West-Nile virus disease related web activities. Autocorrelation function values outside of the two-standard-error bands given by the black lines are statistically significant.

**Fig. 4:**
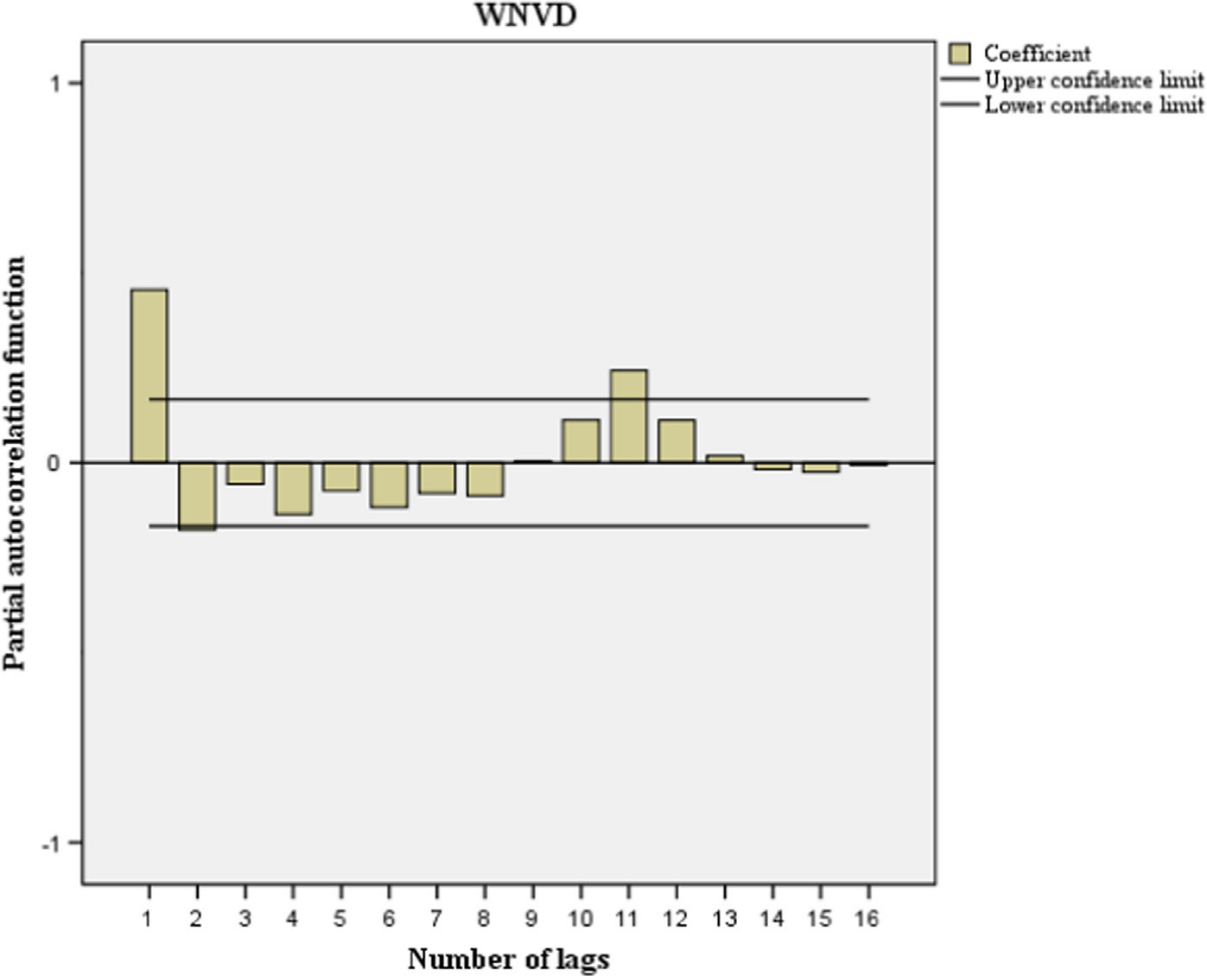
Partial auto-correlation of the Google Trends-generated data concerning the West-Nile virus disease related web activities. Partial autocorrelation function values outside of the two-standard-error bands given by the black lines are statistically significant.

**Fig. 5:**
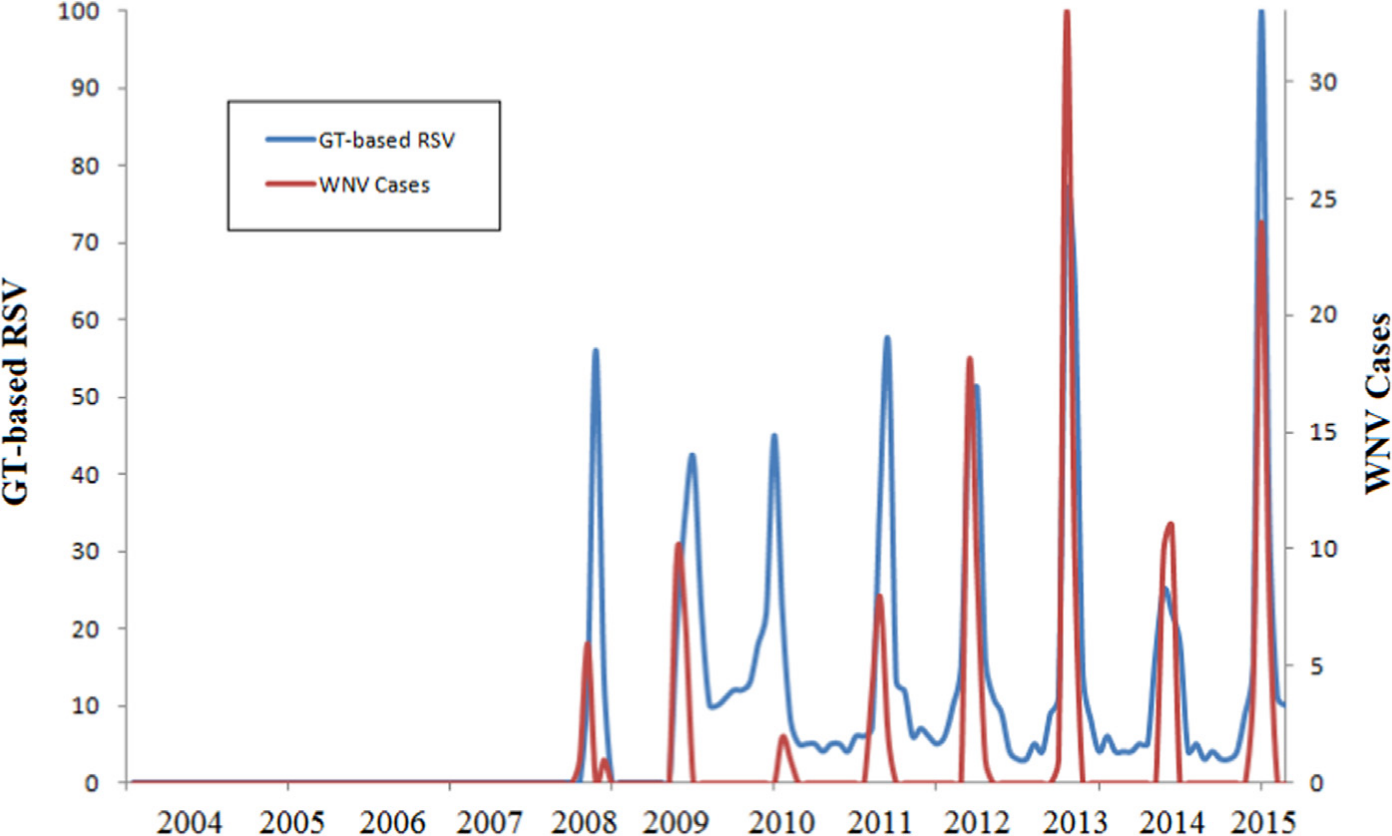
Correlational analysis between the Google Trends-generated data concerning West-Nile virus disease related web activities and the real epidemiological cases.

This paper contains infodemiological data concerning the West-Nile virus diseases related web-activities carried out in Italy from 2004 to 2015 (Fig. 1, Table 1). These data showed a cyclic regular pattern (Fig. 2, Fig. 3, Fig. 4, Table 2, Table 3), well correlating with epidemiological data (Fig. 5, Table 4).

## 2 Experimental design, materials and methods

Google Trends (GT, a tool freely available at https://www.google.com/trends) was mined from 2004 to 2015, searching for West-Nile virus disease (WNVD).

Epidemiological data were obtained and downloaded from the Epicentro Italian National Health Institute (ISS) website (accessible at http://www.epicentro.iss.it/problemi/westNile/Rizzo2011.asp) and from the IZSAM Caporale Teramo website (http://sorveglianza.izs.it/emergenze/west_nile/emergenze.html).

GT-generated data were modeled as a time series and analyzed using classical time series analyses. In order to detect regular time patterns, spectral analysis was carried out using algorithms written in Matlab, freely available at http://paos.colorado.edu/research/wavelets/ [1]. Further, correlation between GT-based Relative Search Volumes (RSVs) related to WNVD and “real-world” epidemiological cases in the same study period was performed both on a monthly basis and on a yearly basis. Correlation between GT-based RSVs related to WNVD was also carried out on a regional basis. Autocorrelation and partial autocorrelation functions enable to compute the correlation of a time series with its own lagged values, respectively non controlling and controlling for the values at all shorter lags. Moreover, a regression model of the GT-generated data concerning WNVD-related web activities was performed.

Autocorrelation and partial autocorrelation analyses, correlational analysis and regressions were performed using the commercial software Statistical Package for Social Science (SPSS, version 23.0, IL, USA) and the commercial software MedCalc Statistical Software version 16.4.3 (MedCalc Software bvba, Ostend, Belgium; https://www.medcalc.org; 2016).

Figures with a *p*-value <0.05 were considered statistically significant.

## Conflicts of interest

All authors declare no conflicts of interest.

## Figures and Tables

**Table 1:** Digital interest for West-Nile virus disease in Italy at regional and town level. Abbreviation: RSV (relative search volume).

**Interest at regional level**	**RSV (%)**	**Interest at town level**	**RSV (%)**
Sardinia	100	Cagliari	100
Emilia-Romagna	60	Bologna	43
Veneto	47	Padua	38
Friuli Venezia Giulia	47	Milan	20
Umbria	32	Rome	20

**Table 2:** Autocorrelation analysis of the Google Trends-generated data concerning West-Nile Virus disease related web activities.

**Lag**	**Autocorrelation**	**Standard deviation**	**Box-Ljung statistics**		
			**Value**	**Degrees of freedom**	**Sig.**
1	0.456	0.082	30.589	1	0.000
2	0.069	0.082	31.284	2	0.000
3	−0.087	0.082	32.400	3	0.000
4	−0.185	0.082	37.536	4	0.000
5	−0.198	0.081	43.471	5	0.000
6	−0.199	0.081	49.504	6	0.000
7	−0.169	0.081	53.899	7	0.000
8	−0.129	0.080	56.467	8	0.000
9	−0.030	0.080	56.610	9	0.000
10	0.139	0.080	59.653	10	0.000
11	0.348	0.080	78.764	11	0.000
12	0.366	0.079	100.104	12	0.000
13	0.207	0.079	107.001	13	0.000
14	0.026	0.079	107.113	14	0.000
15	−0.107	0.078	108.980	15	0.000
16	−0.159	0.078	113.131	16	0.000

**Table 3:** Partial autocorrelation analysis of the Google Trends-generated data concerning the West-Nile virus disease related web activities.

**Lag**	**Partial autocorrelation**	**Standard deviation**
1	0.456	0.083
2	−0.176	0.083
3	−0.056	0.083
4	−0.136	0.083
5	−0.074	0.083
6	−0.118	0.083
7	−0.081	0.083
8	−0.088	0.083
9	0.005	0.083
10	0.113	0.083
11	0.242	0.083
12	0.113	0.083
13	0.019	0.083
14	−0.017	0.083
15	−0.025	0.083
16	−0.007	0.083

**Table 4:** Regression model of the Google Trends-generated data concerning West-Nile virus disease related web activities.

**Indipendent variable**	**Coefficient**	**Standard error**	***r***_**partial**_	***t***	***p*-Value**
Cases	2.55	0.20	0.74	12.87	0.0000
Month	0.75	0.24	0.26	3.18	0.0018
Year	1.00	0.24	0.33	4.17	0.0001
